# Supplementary material for: The Role of Thickness Control and Interface Modification in Assembling Efficient Planar Perovskite Solar Cells
Source: Molecules. 2019 Sep 24;24(19):3466. doi: 10.3390/molecules24193466 (PMC6803942; doi:10.3390/molecules24193466)
Supplement: Supplementary file 1 [file molecules-24-03466-s001.pdf]

The role of thickness control and interface modification in assembling efficient planar perovskite solar cells

Weifu Sun, Kwang-Leong Choy*, MingqingWang

UCL Institute for Materials Discovery, University College London, Roberts Building, Malet Place, London, WC1E 7JE, United Kingdom.

*Corresponding author: ~~yhsu@cc.nctu.edu.tw~~; k.choy@ucl.ac.uk

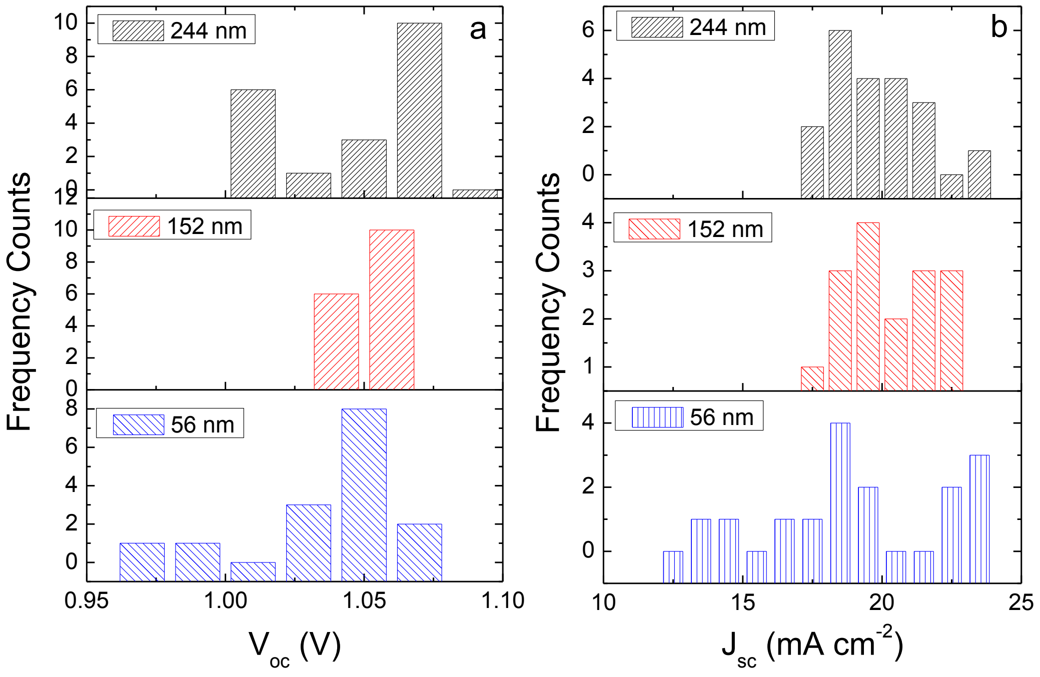

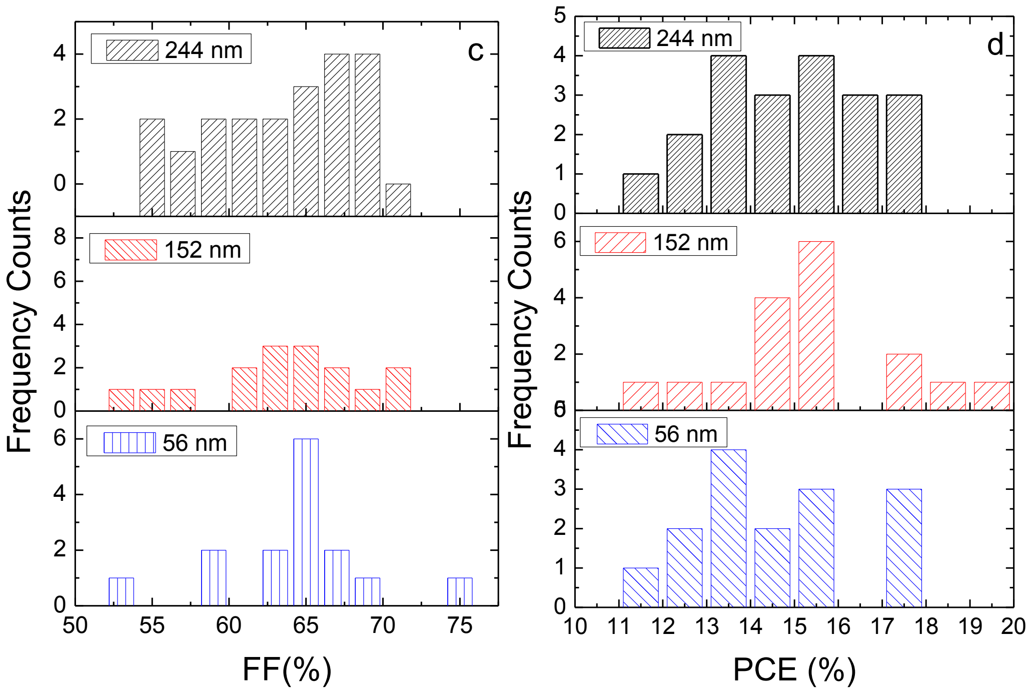

Figure S1 Statistics of photovoltaic performance of the assembled 15 ~ 20 planar PSCs with a typical configuration of FTO/TiO_2/$MAPbI_3$/Spiro-MeOTAD/Ag as a function of thickness of electron transport layer (ETL) TiO_2: (a) V_{oc}, (b) J_{sc}, (c) FF and (d) PCE.

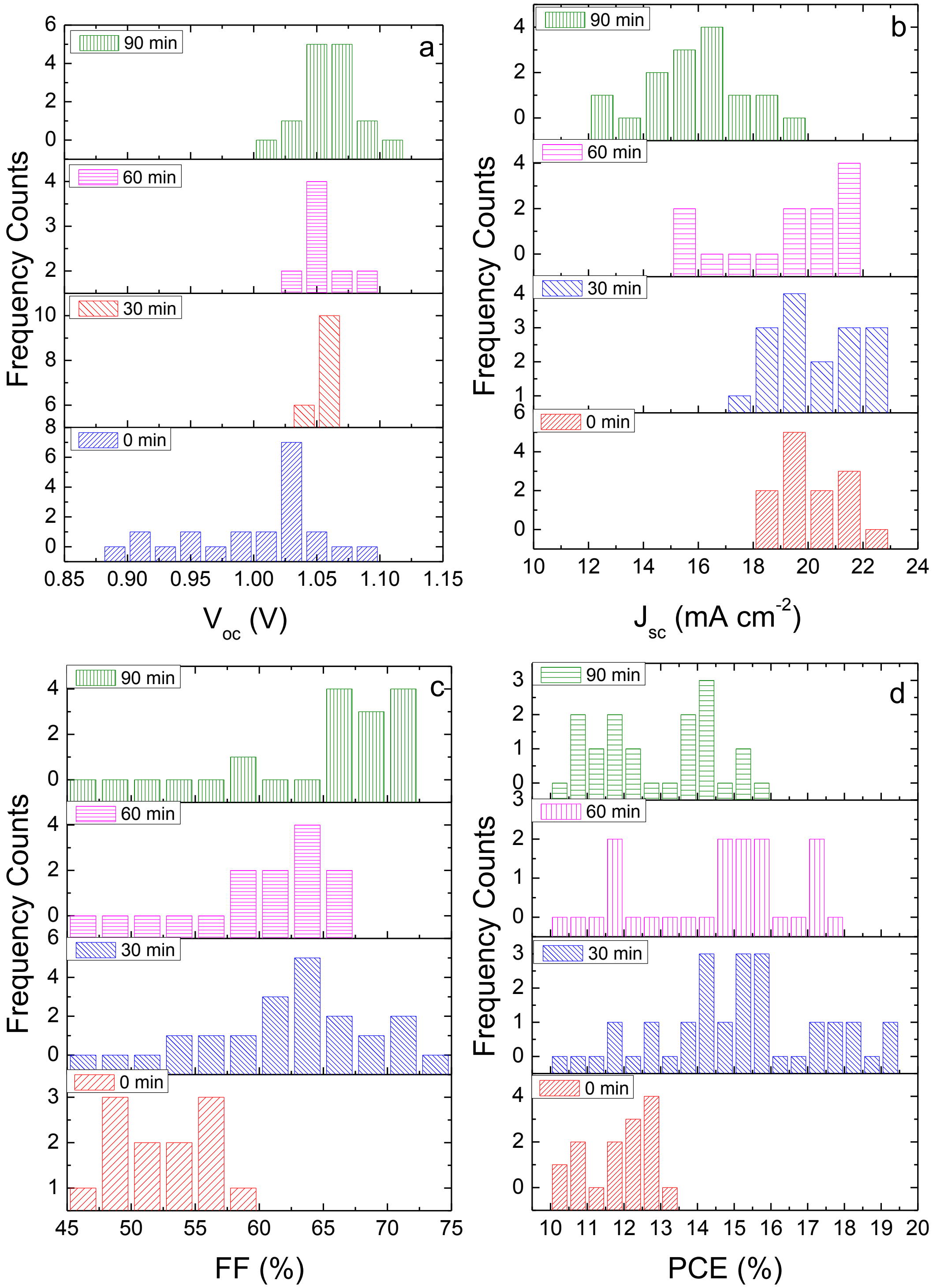

Figure S2 Statistics of photovoltaic performance of the assembled planar PSCs with a typical configuration of FTO/TiO_2/$MAPbI_3$/Spiro-MeOTAD/Ag as a function of different treatment times of $TiCl_4$: (a) V_{oc}, (b) J_{sc}, (c) FF and (d) PCE.

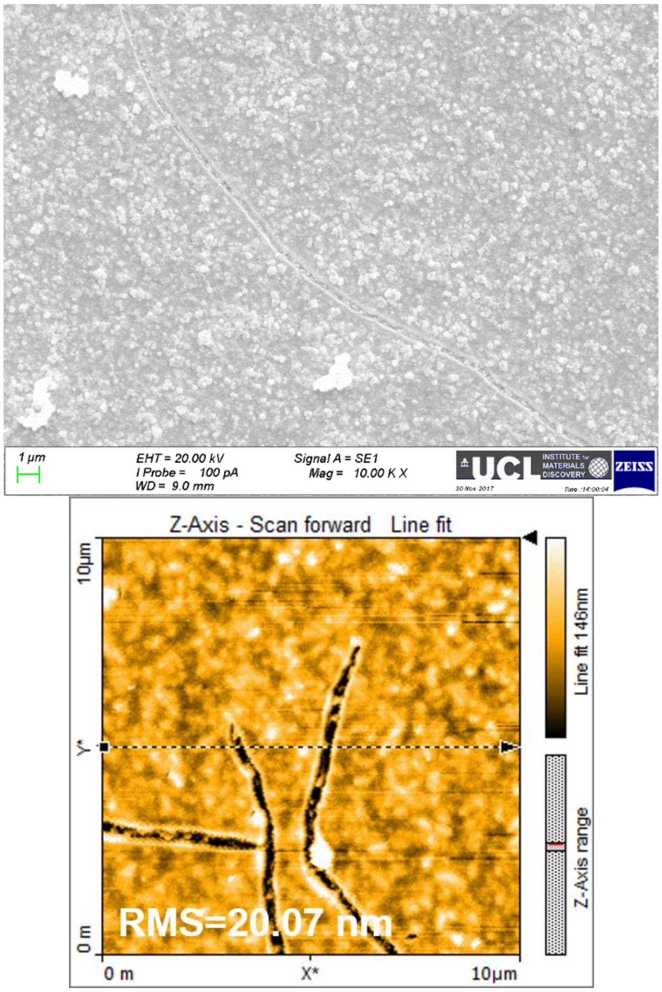

Figure S3 SEM and AFM images of blocking layer TiO_2 after being treated using 40 mM $TiCl_4$ aqueous solution for 30 min.
